# Comparison of the analgesic effect of intravenous paracetamol/midazolam and fentanyl in preparation of patients for colonoscopy: A double blind randomized clinical trial

**Published:** 2015

**Authors:** Abbasali Ahmadi, Parviz Amri, Javad Shokri, Karimollah Hajian

**Affiliations:** 1 Department of Internal Medicine, Babol University of Medical Science, Babol, Iran.; 2 Department of Intensive Care, Babol University of Medical Sciences, Babol, Iran.; 3 Department of Social Medicine and Health, Babol University of Medical Sciences, Babol, Iran.

**Keywords:** Paracetamol, Fentanyl, Colonoscopy, Analgesia

## Abstract

**Background:**

Although some patients can tolerate colonoscopy procedure using fentanyl/ midazolam without any sedation and analgesic requirements but some patients may require additional sedation with benzodiazepines. We performed the present study to compare the effect of paracetamol/midazolam with fentanyl/ midazolam.

**Methods:**

In a clinical trial, 96 patients aged 18 to 75 years old, who were candidate for elective colonoscopy assigned consecutively into two groups as paracetamol/midazolam and fentanyl/midazolam. The first group received 1 gr paracetamol 45 minutes before colonoscopy and 0.5 mg/kg midazolam 5 minutes before colonoscopy whereas the second group received 04- 0.5-1 mcg/kg fentanyl 3 minutes before colonoscopy and similar dose of midazolam. The two groups were compared in regard to patient intensity, discomfort, acolonoscopist and, patient satisfaction and rescue dose of propofol during colonoscopy and vital signs.

**Results:**

There was no significant difference between the two groups for patient pain score, colonoscopist satisfaction, patient satisfaction and rescue dose of propofol (P=0.817, 0.978, 0.460, and 0.104, respectively). The incidence of apnea was greater in fentanyl group (P=0.045). After adjusting for age and education, there was also no significant difference between the two groups.

**Conclusion:**

This study indicates that paracetamol can be considered as an alternative drug regimen in preparation of colonoscopy.

Nowadays colonoscopy is the standard procedure for diagnosis, screening, treatment and follow-up for many colorectal diseases. Although some patients can tolerate colonoscopy procedure without any sedation and analgesic requirements, however, using this drug in some patients is associated with stress (1). There are difficulties in the determination of an optimal dose for sedation and monitoring patients adequately during the procedure. Many patients require intravenous benzodiazepines and opiates (2). These medications are associated with amnesia, anxiolytic, and sedative properties. In addition, combination of benzodiazepine and opioid is associated with several undesirable effects, including a delay of several minutes from the time of injection before the drugs exert their effects, amnesia and risk of respiratory depression (3-5).

Paracetamol is a non-opioid agent, and it is believed that it primarily affects the central nervous system via central cyclooxygenase inhibition, and probably has an indirect influence on the serotoninergic system. Paracetamol has a good safety profile and easily passes through the brain barrier, which is considered as an effective analgesic (6). Intravenous paracetamol has been approved by the FDA for the treatment of mild to moderate pain, as an adjunct to opioid analgesics in the treatment of moderate to severe pain and as well as antipyretic. Intravenous acetaminophen is well tolerated (7-8).

Because of its efficacy, safety, lack of clinically significant drug interactions, and lack of the adverse effects associated with other analgesics, IV acetaminophen is an attractive component of a multimodal analgesic treatment plan (9). In a study by Shening et al., the efficacy of fentanyl and oxycodone-acetaminophen in elderly patients was compared with painless colonoscopy under propofol anesthesia and the results indicated that oxycodone-acetaminophen was safer and more efficient in elderly patients with painless colonoscopy under propofol anesthesia (10). Meta-analysis of the efficacy of acetaminophen for the prevention or treatment of postoperative pain revealed that intravenous acetaminophen was superior to placebo (11). The major concern about standard procedural sedation for colonoscopy is the adverse reaction of drugs used for sedation such as respiratory depression, hypotension and bradycardia. The aim of this study was to compare the efficacy of paracetamol/midazolam versus fentanyl/ midazolam in the preparation of patients for elective colonoscopy.

## Methods

The study was a randomized, double blind, prospective study of 96 patients who presented to an outpatient clinic for colonoscopy. Sample size was estimated based on SD of 2.5 for detection of 1.5 pain score difference between the two groups with 95% confidence level and 80% power. All study subjects gave a written consent for their participation in the study and the study protocol was approved by the Ethics Committee of the Vice-Chancellery for Research of Babol University of Medical Sciences (3326) and IRCT registration number: IRCT:201311227752N5.

The patient candidates for colonoscopy were consecutively assigned into two groups by a trained nurse as group paracetamol/midazolam and group fentanil/ midazolam. Inclusion criteria were: age between 18-75 and ASA class ≤2 (*ASA class. Adapted from) *(12). Exclusion criteria were: history of colonic or rectal resection, neurologic deﬁcit, pregnancy, inability or unwillingness to give informed consent, inpatient status, known hypersensitivity to any of the study medications, acute gastrointestinal bleeding, ASA class 3 or higher, short thick neck, or desire to have colonoscopy without sedation, liver disease (Child-Pugh classification C ), history of large-bowel surgery, psychiatric/emotional disorder, history of addiction to opiates and/or sedatives and poor bowel preparation. Ten patients were excluded from study because of their failure to comply with the age criteria or poor bowel preparation.

All patients received normal saline (100cc) over 15 minutes about 30 – 45 minute before colonoscopy. For group A, the normal saline contained 1000 mg paracetamol. Midazolam (0.05 mg/kg maximum 2.5 mg) infused to all patients 5 minutes before colonoscopy. Fentanyl was infused to group B, two to three minutes before colonoscopy with a dose of 0.5 – 1 mcg/kg. Group A received normal saline with syringe of same volume two to three minutes before colonoscopy. All syringes were coded by anesthetic nurse. Internist resident, colonoscopist, assistant nurse, anesthesiologist, and patient were blinded to medicine type. If a patient had severe discomfort during colonoscopy, propofol was prescribed as rescue bolus dose (0.25-0.5 mg/kg) and repeated if needed.

**Figure 1:**
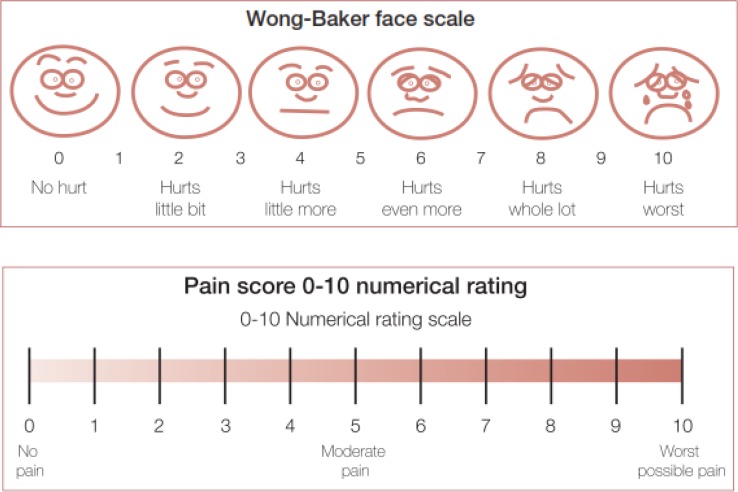
Faces pain scale (FPS

The quality of analgesia and patient satisfaction was assessed using a numerical rating scale (figure 1). Colonoscopy was allowed when Ramsey sedation scale (Ramsey Sedation Scale* Adapted from) *(13) reached to score of 2 or higher after prescribing midazolam. Vital signs (BP, HR, RR, and Sao2) were recorded before initiating the first drug and then every 5 minutes during colonoscopy and in recovery room. Times were recorded by chronometer: insertion of colonoscope, reaching to secum, withdrawing from anus. Colonoscopist satisfaction was assessed after each colonoscopy. Patient pain and satisfaction were assessed when Aldrete score during recovery period reached to score of 9 or higher. We also evaluated the patient discomfort during colonoscopy by faces pain scale (FPS) (figure 1). Episodes of nausea and vomiting were measured during colonoscopy and recovery time. On the basis of recorded items, we measured hypotension as equal or greater than 20 mmHg decrease in systolic blood pressure and/or equal or greater than 10 mmHg decrease in diastolic blood pressure in post infusion period of medicines in proportion to the first recorded BP before initiating the medicine and measured apnea/hypopnea as respiratory rate < 8 for more than 10 seconds and decreased Sao2, classified as Sao2 ≤ 88%, 88%-94% or ≥ 94% in post infusion period of medicines. The amount of rescue doses of propofol was also compared between two groups. We used two independent two-sample t-test for the comparison of the mean pain score between two groups in bivariant analysis. We also used multiple linear regression analysis to adjust the differences of age and education levels for pain score. We estimate the age-education adjusted regression coefficient as mean difference between two groups for comparison.

*Adapted from: *Acute Pain Management Measurement Tool kit; Published by Rural and Regional Health and Aged Care Services Division Victorian Government Department of Human Services Melbourne Victoria Australia February 2007. Downloaded from the VQC website at www.health.vic.gov.au/qualitycouncil

## Results

A total of 42 patients made up the paracetamol group and 44 patients in fentanyl group. There were no significant differences between the groups regarding sex, body weight, ASA physical status, diagnosis and baseline hemodynamic parameters except age and education level (table 1). 

There was no significant difference between the two groups for patient pain score, colonoscopist satisfaction, patient satisfaction, rescue dose and secal time, respectively (P=0.699, 0.969, 0.358, 0.104, 0.605) (table 2). Patient discomfort during colonoscopy was greater in paracetamol group (P=0.036 CI: 0.06–1.6).

 The incidence of hypotension was greater in fentanyl group but not significantly P=0.063 (table 3). Incidence of apnea was greater in fentanyl group (p=0.045) (table3). There was no any nausea and vomiting in two groups. After the age and education adjusted analysis, there was no significant difference in outcome variables between two groups (table 4).

**Table1:** Clinical and demographic characteristics of two groups under study

**Pvalue**	**Fentanyl group**	**Paracetamol group**	**Characteristics**
<0.001	49±11.6	40.10±12.0	age
0.131	25.70±3.68	27.05±4.51	BMI
0.035	37.7	27.15	Education
0.261	91.65±12.61	88.87±9.91	MAP
0.289	18M & 26F	22M &20F	sex
	All patient>95%	All patient >95%	Sao2
0.395	1.38 ± 0.58	1.28±0.50	ASA class

**Table 2:** The comparisons of pain score (analgesic effect) and other clinical outcomes between parcetamol and fentanyl group during colonoscopy

**Independent Samples Test**	**N**	**Mean±SD**	**pvalue**
Patient pain (score)			
Paracetamol Fentanyl	42 44	4.00±2.632 3.77±2.786	0.699
Colonoscopist consent (score)			
Paracetamol Fentanyl	42 44	7.76±1.246 7.75±1.557	0.969
Patient consent (score)			
Paracetamol Fentanyl	40 39	9.35±0.975 9.13±1.151	0.358
Rescue dose (mg)			
Paracetamol Fentanyl	42 44	19.55±19.848 13.41±14.458	0.104
Patient discomfort (score)			
Paracetamol Fentanyl	42 44	6.36±1.511 5.52±2.063	0.036
Secal Time (second)			
Paracetamol Fentanyl	37 41	350.24±126.543 336.29±110.874	0.605

**Table 3:** Comparing the adverse effect of parcetamol and fentanyl during colonoscopy*

	**Group**		**p-value**
	**paracetamol**	**fentanil**	
Hypotension			
Sig.decreas No sig decrease Total	11 (26.2%) 31 (73.8%) 42	20 (45.5%) 24 (54.5%) 44	0.063
apnea/hypopnea			
yes no total	0 (0%) 42 (100%) 42	4 (9.1%) 40 (90.9%) 44	0.045
Forget			
Yes No Total	7 (17.1%) 34 (82.9%) 41	9 (22%) 32 (78%) 41	0.577
Sao2			
<88% 88-94% >94% total	0 (0%) 8 (19%) 34 (81%) 42	4 (9.1%) 10 (22.7%) 30 (68.2%) 44	0.109

Sao2: arterial o2 saturation. sig. decrease: significant decrease in blood pressure.

Forget: Inability to remember what happened during colonoscopy

* By chi-square test

**Table 4:** Age and education adjusted regression coefficient fentanyl vs paracetamol and its SE and p-value

**Dependent variable**	**Coefficient(B)** *	**SE** **	**pvalue**
Patient pain score	-0.06	0.61	0.92
Colonoscopist consent score	-0.10	0.33	0.76
Patient consent score	-0.25	0.27	0.35
Rescue dose	-4.22	4.07	0.30
Patient discomfort	-0.74	0.42	0.08
Secal time	-23.70	29.02	0.42
Apnea	0.10	0.05	0.05
Hypotension	-0.16	0.11	0.15
Fullness	0.04	0.10	0.67

Linear regression analysis

* Coefficient: adjusted mean difference between two groups

** SE: standard error

## Discussion

Paracetamol is a viable alternative to the NSAIDs in postoperative pain management, especially because of the low incidence of adverse effects, and should be a preferred choice in high risk patients (14). Several studies have noted paracetamol clinical beneﬁts by providing reduced pain scores, opioid consumption, and postoperative side effects when used as a postoperative analgesic (15). In the immediate period after ambulatory parathyroidectomy (0–30 min), pain scores were not signiﬁcantly different between the ketorolac and intravenous acetaminophen groups; however, pain scores were signiﬁcantly lower in the later postoperative period (45, 60 and 75 min) in the group of patients who received ketorolac (16). Although paracetamol (1gr) has caused a better pain relief quality but it is not a suitable analgesic for moderate pain control in acute phase after laparoscopic cholecystectomy alone (17). Preemptive intravenous paracetamol produces significant opioid sparing effects compared to placebo in postoperative patients following cholecystectomies. It decreased 24 h total opioid consumption and increases the time for the first analgesic use, thus, its analgesic effect was not enough as a sole agent (18). Endoscopic sinus surgery is associated with significant postoperative pain. Acetaminophen provides adequate pain relief in most patients who have undergone ESS. However, the analgesic efficacy of acetaminophen alone is insufficient in some patients, and hence, all patients with ESS must be followed closely to identify those patients in need of more efficient analgesia during the early phase of recovery (19). Ali M. and Khan FA. Assessed postoperative pain with a visual analog scale (VAS), which is a valid tool for the measurement of pain but has certain limitations. A VAS measures pain as a unidimensional experience. It quantifies only the intensity of pain and not the quality of pain. Patients may vary randomly in how they place their mark on the scale. VAS is not easily administered to patients who have perceptual motor problems (20). 

In the literature review, we found one study (Shen et al.) concluded that oxycodon-acetaminophen is safer and more effective than fentanyl in old patients during colonoscopy, while we resulted that intravenous paracetamol is comparable to fentanyl in analgesic effect during colonoscopy. The conclusions in our study and study of Shen et al. is somewhat comparable.

Colonoscopy is a painful and unpleasant procedure for many patients. Therefore, opiates, benzodiazepines, and propofol in various combinations are administered to these patients to provide sedation, analgesia/sedation, or general anesthesia (21). All these drugs have side effects such as CNS and respiratory suppression and hemodynamic compromise that could potentially be especially in elderly or patients with cardiovascular, respiratory or central nervous system (CNS) disease. 

Therefore, an analgesic without sedative effects might be useful in mentioned situation. This study showed that paracetamol/midazolam is comparable with fentanyl/ midazolam to reduce patient pain during colonoscopy. Although the sample size in this study was not large enough to detect the differences in rates of complications such as apnea, but the incidence of apnea and hypotension appears to be lower in group paracetamol. We suggest that in situation in which higher incidence of CNS and respiratory suppression is expected, paracetamol could be a good alternative for opioid in colonoscopy especially if an anesthesiologist is not present. 

One limitation of paracetamol use in colonoscopy is the time paracetamol required to reach its maximum analgesic effect. This can lead to delays in doing the colonoscopy. Further work needs to be done to determine the best time to start colonoscopy after administration of intravenous paracetamol. The sample size in this study was not large enough to detect the differences in rates of complications such as apnea, the incidence of apnea and hypotension appears to be lower in paracetamol group.

In this study, we concluded that the analgesic effect of paracetamol is comparable to fentanil in patient during colonoscopy under midazolam sedation. We suggest that in situation in which higher incidence of CNS and respiratory suppression is expected, paracetamol could be a good alternative for opioid in colonoscopy especially if an anesthesiologist is not present. 

## Notes

**Funding: **This article is the result of a research project with code number 1934 of Babol University of Medical Sciences.

## Notes

**Conflict Interest: **None declared.
